# Palliative Care bei über 80-jährigen Patienten mit COVID‑19-Pneumonie auf der Intensivstation – invasive Beatmung zielführend?

**DOI:** 10.1007/s10354-022-00917-2

**Published:** 2022-03-22

**Authors:** Dagmar Vohla

**Affiliations:** 1 3. Medizinische Abteilung, Krankenhaus Hietzing, Wien, Österreich; 2 Paracelsus Medical University, Salzburg, Österreich

**Keywords:** COVID‑19, Geriatrische Intensivmedizin, Intubation, Best Supportive Care Scores, COVID‑19, Geriatric intensive care, Intubation, Best supportive care scores

## Abstract

Mit dem Auftreten des neuartigen SARS-CoV-2-Virus im Februar 2020 und der damit assoziierten COVID‑19-Viruspneumonie kam es zu einer Vielzahl schwer erkrankter Patienten auf den Intensivstationen. Gerade zu Beginn der Pandemie zeigte sich eine hohe Mortalität insbesondere der intubierten Patienten. Auch wenn sich durch gewonnene Erfahrungswerte in der Beatmung der schwer kranken Patienten die Mortalitätsrate senken ließ, beträgt sie in der Patientengruppe der über 80-Jährigen weiterhin 80 %. Anhand des vorgestellten Patientenbeispiels wird erörtert, inwieweit validierte Scores unterstützen, eine Entscheidung bezüglich einer möglichen Intubation vs. Best Supportive Care zu finden.

## Abstract (en)

In February 2020 a new virus named-SARS-CoV‑2 emerged. As a result many patients suffered from COVID‑19-associated pneumonia and were in need of hospitalization in an intensive care unit (ICU). Especially at the beginning of this pandemic the mortality among intubated patients was very high. With ongoing experience in how to handle COVID‑19-associated respiratory insufficiency the mortality rate was reduced but is still high among older patients. In this case report it is discussed if validated scores can support the decision between intubating patients or providing best supportive care.

Das Auftreten des neuartigen SARS-CoV-2-RNA-Virus zu Beginn des Jahres 2020 hat auch die Intensivmedizin vor neue Herausforderungen gestellt. Die intensivmedizinisch zu betreuenden Patienten, 3 % der an COVID‑19 Erkrankten, zeichnen sich durch respiratorische Insuffizienz mit bilateraler Pneumonie im Sinne eines Acute-Respiratory-Distress-Syndroms (ARDS) aus [1]. Beim Großteil dieser Patienten besteht bereits früh Sauerstoffbedarf. Um den zehnten Tag nach Symptombeginn kommt es meist zu einer nochmaligen deutlichen respiratorischen Verschlechterung.

Charakteristisch bei dieser Viruspneumonie ist eine in den Blutgasen deutlich sichtbar gemessene Hypoxämie bei oft fehlender Dyspnoesymptomatik [1]. Bei insuffizienter Beatmung durch eine „high flow nasal cannula“ (HFNC) oder eine non-invasive Ventilation(NIV)-Maske ist eine Intubation notwendig. Im Vergleich zu Krankheitsverläufen bei bakteriellen Pneumonien ist die COVID‑19-Pneumonie durch eine lange Intubationsdauer mit hohem Beatmungsaufwand gekennzeichnet [1]. Dies stellt bei bereits verminderten körperlichen Ressourcen wie hohem Alter und Vorerkrankungen ein entscheidendes Problem dar. Ein positives Outcome ist durch diese Faktoren deutlich reduziert.

Auffällig zeigt sich nach unserer Erfahrung die Notwendigkeit hoher Sedierungsdosen. Um die Bauchlagerung, welche zu einer besseren Oxygenierung führt, zu gewährleisten, werden die Patienten zusätzlich relaxiert. Das Sedierungsregime kann wiederum besonders bei älteren Patienten vermehrt zum Auftreten eines Delirs, einer verzögerten Aufwachreaktion und einer Critical-Illness-Neuropathie führen [2]. In einer hohen Zahl der Fälle ist aufgrund der langen Intubationsdauer und des oftmals schwierigen Weanings eine Tracheotomie notwendig.

Bei ausreichendem Vorhandensein intensivmedizinischer Betten auf unserer internistischen Intensivstation, die seit Beginn der Pandemie im März 2020 mit ca. 170 Patienten fast ausschließlich COVID‑19-Patienten betreut hat, bestand bisher keine Notwendigkeit einer aktiven Triage von Patienten. Folglich wurde von einer Intubation nur bei ausdrücklichem Wunsch des Patienten und ausgeprägter Multimorbidität Abstand genommen.

Infektionen mit COVID‑19 führen bei Patienten im hohen Alter auch bei wenigen Vorerkrankungen zu einem oft tödlichen Verlauf. Hinzu kommt, dass sich in Studien zeigt, dass bereits ein Alter von > 75 Jahren bei invasiv beatmeten Patienten einen unabhängigen Risikofaktor für ein schlechtes Outcome darstellt [3]. Somit stellt sich die Frage, ob eine Intubation bei nahezu 100 % Mortalität bei über 80-Jährigen mit COVID‑19-Pneumonie und vorbestehenden Komorbiditäten eine zielführende Therapie darstellt. In diesem Zusammenhang gibt es die Überlegung des Einsatzes von Scores, die eine Aussagekraft bezüglich des Outcomes dieser Patienten haben und die bei der Entscheidungsfindung unterstützen können.

## Patientenbeispiel

Zur Aufnahme auf die Normalstation kam Herr M., 79-jährig, zu Hause mit der Ehefrau selbstständig lebend. Als Vorerkrankung bestand ein nicht insulinpflichtiger Diabetes mellitus Typ 2.

Herr M. wurde mittels PCR-Test positiv auf SARS-CoV‑2 getestet mit initialen Symptomen einer Diarrhö und Müdigkeit. Aufgrund einer Erhöhung des D‑Dimer wurde bereits auf der Notfallabteilung zum Ausschluss einer Pulmonalembolie eine Computertomographie des Thorax durchgeführt, in der sich Veränderungen im Sinne einer Viruspneumonie zeigten.

Bei steigendem Sauerstoffbedarf kam es nach 5 Tagen zur Transferierung auf die Lungenabteilung (Intermediale Care) zur weiteren Therapie mit HFNC und in weiterer Folge mit „continuous positive airway pressure“ (CPAP). Im Verlauf-Thoraxröntgen zeigte sich eine Dichtezunahme der pneumonischen Infiltrate. Sechs Tage später kam es zu einer weiteren Verschlechterung, und eine ausreichende Oxygenierung mittels HFNC/CPAP konnte nicht mehr erreicht werden, sodass Herr M. am 15. Tag nach COVID-Symptombeginn auf die Intensivstation verlegt wurde. Es erfolgte nach Aufnahme die rasche Intubation mit Bauchlagerung.

Bei steigendem Procalcitonin wurde eine antimikrobielle Therapie initiiert. Ab dem 8. Tag nach Intubation kam es zu einer Besserungstendenz bezüglich der Beatmungseinstellungen, sodass mit dem Weaning begonnen wurde. Allerdings zeigte sich nach Beendigung der Sedierung keine adäquate Aufwachreaktion des Patienten. Es erfolgte eine Computertomographie des Schädels mit Angiographie der Hirngefäße, in dem sich kein Hinweis auf eine Ischämie oder Blutung zeigte. Auch in dem in weiterer Folge durchgeführten Elektroenzephalogramm und einer kranialen Magnetresonanztomographie wurde kein ursächlich fassbares Korrelat für die verminderte Vigilanz gefunden.

Da sowohl aufgrund der bestehenden Beatmungseinstellung und der neurologischen Situation eine Extubation nicht möglich war, erfolgte eine Tracheotomie.

Medikamentös wurde die Behandlung sowohl eines hypoaktiven Delirs als auch einer Critical-Illness-Myopathie/Polyneuropathie initiiert.

Im weiteren Verlauf kam es undulierend zu einer minimalen Vigilanzbesserung mit Augenöffnung und Blickfolge. Intermittierend konnte für ein paar Stunden die Beatmung auf HFNC umgestellt werden, jedoch musste der Patient nach respiratorischer Erschöpfung wieder an die Beatmungsmaschine genommen werden. Da sich in den 2 darauffolgenden Wochen bei protrahiertem Verlauf und beginnendem Multiorganversagen unter Ausschöpfung der intensivmedizinischen Maßnahmen keine Besserung zeigte, wurde schließlich nach Beschluss im intensivmedizinischen Team eine palliative Behandlungsstrategie eingeschlagen. Der Patient verstarb am 41. Tag nach Aufnahme auf der Intensivstation und 57 Tage nach Infektionsbeginn.

## COVID‑19 und Intubation

Den Erfahrungen nach und auf Basis zahlreicher Studien, die in der Zeit der ersten Welle erfolgten, wird in der Zwischenzeit versucht, die respiratorische Insuffizienz möglichst lange mittels HFNC und NIV-Beatmung zu behandeln und eine Intubation, wenn möglich, zu vermeiden [1]. Als Leitlinie zur sofortigen Intubation gelten eine schwere Hypoxämie bei p_a_O_2_/F_i_O_2_ < 100 mm Hg, in Erwägung sollte eine Intubation gezogen werden bei p_a_O_2_/F_i_O_2_ < 150 mm Hg und Atemfrequenzen > 30/min [4].

Verschiedene Studien zeigten unterschiedliche Empfehlungen bezüglich einer frühzeitigen oder einer verzögerten Intubation [4, 5]. Die Determinante für das Outcome scheinen die Schwere und der Fortschritt der Erkrankung zu sein. In Bezug auf Letzteres ist es wichtig, nicht außer Acht zu lassen, dass es durch eine bereits mehrere Tage bestehende nichtinvasive Beatmung zu einer respiratorischen Erschöpfung v. a. der Atemmuskulatur kommen kann. Seitens mancher Patienten besteht allerdings eine erstaunliche Symptomlosigkeit („happy Hypoxia“). Die Erschöpfung macht sich nach erfolgter Intubation durch eine deutlich verlängerte Rekompensationszeit der respiratorischen Situation bemerkbar.

Eine Reduktion der Beatmungseinstellungen ist langwierig, sodass nach einer ca. 14-tägigen Intubation eine Tracheotomie erfolgt [4].

Unabhängig von der Pandemie zeigt sich in den letzten Jahren ein Anstieg der über 80-jährigen Patienten auf den Intensivstationen mit einem Prozentsatz zwischen 15 und 30 % weltweit [6]. Auch bei anderen intensivpflichtigen Krankheitsbildern besteht die Überlegung, wie sinnvoll eine intensivmedizinische Behandlung in diesem Alter noch ist. Es kann festgehalten werden, dass unabhängig vom Alter für die Entscheidung zur Aufnahme oder Ablehnung einer intensivmedizinischen Maßnahme weitere Faktoren wie der funktionale Aktivitätszustand, der Schweregrad der Organdysfunktion, die Prognose der Erkrankung und der Grad an Komorbiditäten mit berücksichtigt werden sollten [6].

Physiologisch zeigt sich mit zunehmendem Alter aufgrund der Abnahme der kognitiven Funktionsreserven mit Reduktion der Hirnmasse und der zerebralen Perfusion die Gefahr einer beschleunigten Entwicklung eines Delirs.

In Bezug auf die Lunge besteht eine verminderte physiologische Reserve, ebenso der Atempumpe und der Atemwegsabwehr mit einem erhöhten Risiko für Atemwegskomplikationen.

Auch bei den restlichen Organsystemen (kardiovaskulär, renal, gastrointestinal, endokrin) besteht eine altersbedingte Verringerung der Funktionsreserve mit erhöhter Gefahr eines Organversagens [6].

## Scores

Eine Unterscheidung in Scores, die eine allgemeine Aussage über das Outcome der Patienten nach intensivmedizinischen Behandlungen voraussagen, und in Scores, die sich spezifisch auf eine Erkrankung beziehen, ist gegeben. In Bezug auf COVID‑19 vermehrt Anwendung findet der CURB-65-Score, spezifiziert als A‑DROP, für COVID‑19, der APACHE II-Score als Vorhersage der Spitalsmortalität und der 4C-Mortality-Score (Tab. 1; [7]). Auf Intensivstationen in Österreich wird der SAP III-Score verwendet, ähnlich zum APACHE II-Score.

**Table 1:** 

Variablen	4C Mortality Score for COVID‑19	
*Alter*		
< 50	–	
50–59	+2	
60–69	+4	
70–79	+6	
> 80	+7	
*Geschlecht*		
weiblich	–	
männlich	+1	
**Anzahl an Komorbiditäten** Komorbiditäten inkludieren chronische Herzerkrankungen, chronische Lungenerkrankungen (Asthma bronchiale nicht inkludiert), chronisch renale Insuffizienz (GFR ≤ 30), leichte bis schwere Lebererkrankungen, chronisch neurologische Erkrankungen, Bindegewbserkrankungen, Diabetes mellitus (Diät, OAD oder Insulin), HIV oder AIDS, und maligne Erkrankungen	0, +1, +2	
**Atemfrequenz/min**	< 20	0
	20–29	+1
	≥ 30	+2
**Periphere Sauerstoffsättigung bei Raumluft**	≥ 92 %	0
	< 92 %	+2
**Glasgow Coma Scale**	15	0
	< 15	+1
**BUN**	< 19,6 mg/dL	0
	≥ 19,6 to ≤ 39,2 mg/dL	+1
	> 39,2 mg/dL	+2
**C‑reaktives Protein**	< 50 mg/L	0
	50–99 mg/L	+1
	≥ 100 mg/L	+2

*GFR* Glomeruläre Filtrationsrate, *OAD* orale Antidiabetika, *BUN* Blut-Harnstoff-Stickstoff

Bei älteren Patienten macht es Sinn unabhängig vom chronologischen Alter mittels Scores eine Einschätzung bezüglich der Funktionalität zu bekommen, da eine erhöhte Einschränkung mit der Mortalitätsrate korreliert [6]. Zur Verwendung kann die Instrumental Activities of Daily Living(IADL)-Skala (Tab. 2) herangezogen werden, welche 8 zentrale Aktivitäten des täglichen Lebens erfasst, und die Clinical Frailty Scale (Tab. 3), bei der durch entsprechende Fragen eine bestehende Hilfsbedürftigkeit in alltäglichen Situationen beurteilt wird. Dies ist insofern relevant, da im Rahmen des Frailty-Syndroms bereits kontinuierliche inflammatorische Prozesse unabhängig von der Grunderkrankung bestehen und es zu Veränderungen in der Blutgerinnung kommt, wodurch sich eine erhöhte Mortalität bei intensivmedizinischer Behandlung vorhersagen lässt [6].

**Table 2:** 

Funktion		Punkte
*Funktion*	Benutzt Telefon aus eigener Initiative	1
	Wählt einige bekannte Nummern	1
	Nimmt ab, wählt aber nicht selbstständig	1
	Benutzt das Telefon gar nicht mehr	0
*Einkaufen*	Kauft selbstständig die meisten Dinge ein	1
	Macht wenige Einkäufe	0
	Benötigt beim Einkaufen Begleitung	0
	Kann nicht einkaufen	0
*Kochen*	Plant und kocht die nötigen Mahlzeiten selbstständig	1
	Kocht nötige Mahlzeiten nur nach Vorbereitung durch Dritte	0
	Kocht selbstständig, hält aber benötigte Diät nicht ein	0
	Benötigt vorbereitete und servierte Mahlzeiten	0
*Haushalt*	Hält Haushalt in Ordnung bzw. benötigt Assistenz bei schweren Arbeiten	1
	Führt selbstständig kleine Hausarbeiten aus	1
	Kann kleine Hausarbeiten ausführen, aber nicht die Wohnung reinhalten	1
	Benötigt Hilfe in allen Haushaltsverrichtungen	1
	Kann keine täglichen Arbeiten im Haushalt mehr ausführen	0
*Wäsche*	Wäscht sämtliche eigene Wäsche	1
	Wäscht kleine Sachen	1
	Gesamte Wäsche muss fremdorganisiert werden	0
*Verkehrsmittel*	Benutzt unabhängig öffentliche Verkehrsmittel, eigenes Auto	1
	Bestellt und benutzt das Taxi, aber keine öffentlichen Verkehrsmittel	1
	Benutzt öffentliche Verkehrsmittel in Begleitung	1
	In beschränktem Umfang Fahrten im Taxi oder Auto in Begleitung	0
	Benutzt überhaupt keine Verkehrsmittel mehr	0
*Medikamente*	Nimmt Medikamente selbstständig zur richtigen Zeit in richtiger Dosierung	1
	Nimmt vorbereitete Medikamente korrekt	0
	Kann Medikamente nicht mehr korrekt einnehmen	0
*Geldgeschäfte*	Regelt Geldgeschäfte selbstständig (Budget/Überweisungen/Gang zur Bank)	1
	Erledigt täglich kleine Ausgaben; benötigt Hilfe bei Bankgeschäften	1
	Kann nicht mehr mit Geld umgehen	0

**Table 3:** 

Kategorie	Piktogramme	Einstufung	Beschreibung
1	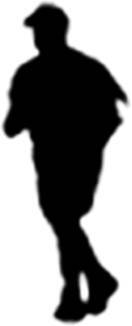	Sehr fit	Robust, aktiv, energisch, gut motiviert und fit: Diese Menschen trainieren regelmäßig und zählen zur fittesten Gruppe in ihrem Alter
2	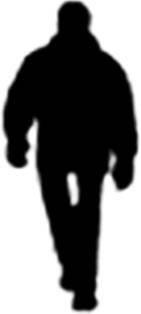	Gut	Ohne aktive Erkrankungen, aber weniger fit als Menschen der Kategorie 1
3	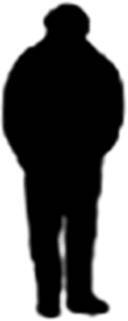	Gut mit behandelten Komorbiditäten	Krankheitssymptome sind im Vergleich zur Kategorie 4 gut kontrolliert
4	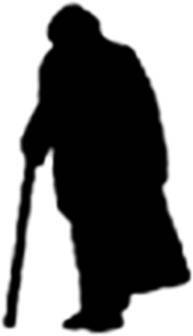	Scheinbar vulnerabel	Obwohl nicht offensichtlich abhängig von anderen Menschen, beklagen sie doch, langsam geworden zu sein und Krankheitssymptome aufzuweisen
5	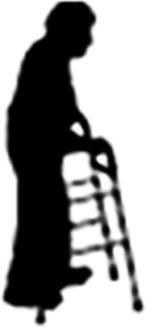	Leicht gebrechlich („frail“)	Mit begrenzter Abhängigkeit von anderen in den instrumentellen Aktivitäten des täglichen Lebens
6	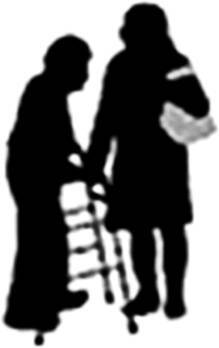	Mittelgradig gebrechlich	Hilfe ist in den instrumentellen und nichtinstrumentellen Aktivitäten des täglichen Lebens nötig
7	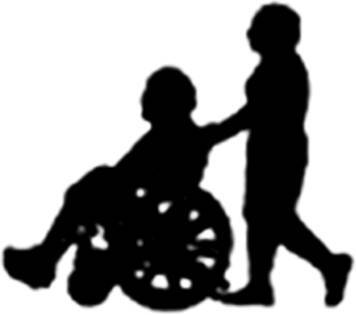	Sehr gebrechlich	Komplett abhängig von anderen Menschen in den Aktivitäten des täglichen Lebens oder im Endstadium krank

*CSHA* Canadian Study on Health and Aging

Die bereits für COVID‑19 erwähnten Scores erfassen wie folgt:

CURB-65: Zur klinischen Einschätzung des Schweregrades einer ambulant erworbenen Pneumonie mit den Parametern: Verwirrtheit („confusion“), Harnstoff-Stickstoff (Urea-N), Atemfrequenz („respiratory rate“), Blutdruck und Alter > 65 Jahre.

APACHE II: Ist ein auf der Intensivstation verwendeter Score, um eine Aussage über die Schwere einer Erkrankung zu machen. Es werden 3 verschiedene Anteile, der „acute physiology score“, die „age points“ und die „chronic health points“ berücksichtigt. In Studien zeigte er sich im Vergleich zum CURB-65 und dem Sepsis-related Organ Failure Assessment(SOFA)-Score, der zur Beurteilung des Zustandes und des Ausmaßes der Organschädigung bei Sepsis auf der Intensivstation verwendet wird, aussagekräftiger bezüglich Spitalsmortalität in Bezug auf COVID‑19 [8].

4C-Mortalitäts-Score: Erlaubt bereits am Aufnahmetag eine Einschätzung des In-hospital-mortality-Risikos mit Unterteilung in niedrig, mittel, hoch und sehr hoch mit zusätzlicher Prozentangabe. Es werden insgesamt 8 Variablen bezüglich Geschlecht, Begleiterkrankungen, Atemfrequenz, periphere Sauerstoffsättigung, Glasgow-Coma-Scale, Harnstoff und C‑reaktives Protein miteinbezogen.

## Conclusio

Die Basis der Therapiezielfindung beruht immer auf dem Patientenwillen und der medizinischen Indikation. Die medizinische Indikation verlangt eine realistische Nutzenerwartung im Rahmen einer vertretbaren Schadensabwägung auf Basis des jeweils aktuellen Erkenntnisstandes [6].

Das Herausfinden des Patientenwillens ist in einer Notsituation nicht immer einfach. Die Deutsche Palliativgesellschaft empfiehlt, bei bereits vorhandenen Patientenverfügungen die Patienten aufzuklären und Zusatzblätter spezifisch für COVID‑19 auszufüllen [9]. Patienten, die bereits auf der Normalstation sauerstoffpflichtig sind, sollten bei einsetzender Verschlechterung über die weiteren möglichen Schritte informiert werden, und der bestehende Patientenwunsch sollte erfasst und dokumentiert werden.

Im vorgestellten Patientenbeispiel hätte sich bei einer Evaluierung mittels Scores bei der primären Aufnahme ein Frailty-Score von 2 bis 3 ergeben, was einen „Gesund“- bis „Angemessen“-Zustand ergibt. In einer deutschen Studie mit 308 ICU-Patienten > 80 Jahre zeigte sich bei einem Wert von 5 („leicht gebrechlich“) ein 30 Tage Mortalitätsrisiko von 31 % [10]. Der 4C-Mortalitäts-Score ergab ein hohes Risiko, schwer an COVID‑19 zu erkranken. verbunden mit einer 30 %igen In-hospital-Mortalität.

Die Problematik zeigte sich an der bereits langen Hospitalisierung mit 14 Tagen vor dem Intensivaufenthalt, sodass es innerhalb dieser Zeit zu einer deutlichen Verschlechterung kam und eine Neuevaluierung der Scores ein wesentlich schlechteres Outcome berechnet hätte. Der Frailty-Score wäre zum Zeitpunkt der Aufnahme auf der Intensiv bei 6 bis 7 gewesen, ebenso der 4C Mortality-Score mit einem Mortalitätsrisiko von 60 %.

In einer Studie zeigt sich, dass die ärztliche Entscheidung bezüglich intensivmedizinischer Maßnahmen meist „intuitiv“ statt „rational“ ist [11]. Der Patient war zu Beginn in einem für sein Alter sehr guten Allgemeinzustand, er wurde intermittierend auf eine andere IMC zur HFNC und NIV-Beatmung verlegt und kam dann direkt auf die Intensivstation. Eine Patientenverfügung gab es nicht, die Transferierung erfolgte bei akut einsetzender massiver Verschlechterung. Einer der limitierenden Faktoren dürfte die lange Intensivpflichtigkeit und protrahierte maschinelle Beatmung gewesen sein, die gerade bei älteren Patienten mit ein wichtiges Outcome-Kriterium ist.

Scores können bei der Entscheidungsfindung ein hilfreiches Tool sein, jedoch sollten sie immer in Kombination mit dem Patientenwunsch und v. a. der klinischen Einschätzung verwendet werden, wobei das Alter nur einen geringen Anteil am prognostischen Teil der Scores hat und nicht als alleiniger Faktor für eine Entscheidungsfindung herangezogen werden kann [12]. Unter diesen Bedingungen können sie als Einbindung nachvollziehbarer Kriterien gemeinsam mit der Einschätzung des Schweregrads der Erkrankung dienen. Verwendung finden sie v. a. dort, wo auch eine Ressourcenknappheit besteht und Allokationsentscheidungen notwendig werden [13].

Beim hier geschilderten Patientenbeispiel hätten sie eine objektive Hilfe zur alternativen Entscheidung einer palliativen Betreuung zum Zeitpunkt der intubationspflichtigen respiratorischen Insuffizienz sein können mit Linderung der vorherrschenden Symptome von Dyspnoe, Fieber, Husten, Schwäche und Delir mittels Morphin-Perfusor und Midazolam-Perfusor.

Ein weiterer v. a. sozialpsychologisch bedeutender Faktor, der nicht unerwähnt bleiben sollte, ist, dass während einer Pandemie Begleitung und Support der Angehörigen aufgrund der erhöhten Infektionsgefahr nicht möglich sind. Auch ein adäquates Abschiednehmen durch die Familie kann nicht stattfinden. Hoffnung allerdings besteht derzeit in den verschiedenen Impfstoffen gegen eine SARS-CoV2 Infektion, sodass die vulnerabelsten Menschen vor schwer verlaufenden COVID‑19-Infektionen geschützt werden können.

Wichtig wäre es, vor allem bei vorbestehender Multimorbidität eine vorzeitige Abklärung des Patientenwunsches durchzuführen, sodass Patienten, die eine Intubation ablehnen, ausreichend palliativ versorgt werden können und für diese Patienten eine Triagesituation vermieden werden kann.
